# 2^3^ Full Factorial Model for Particle Size Optimization of Methotrexate Loaded Chitosan Nanocarriers: A Design of Experiments (DoE) Approach

**DOI:** 10.1155/2018/7834159

**Published:** 2018-09-25

**Authors:** S. P. Surya Teja, N. Damodharan

**Affiliations:** Department of Pharmaceutics, SRM College of Pharmacy, SRM Institute of Science & Technology, India

## Abstract

**Purpose:**

To build and inquire a statistically significant mathematical model for manufacturing methotrexate loaded chitosan nanoparticles (CsNP) of desired particle size. The study was also performed to evaluate the effect of formulation variables in the explored design space.

**Method:**

Ionotropic gelation technique was followed for chitosan nanocarriers by changing formulation variables suggested as per Design Expert software. Altering the levels of Chitosan, tripolyphosphate, methotrexate by 2^3^ factorial design served the purpose. The CsNP were characterized for nanocarrier formation, particle size, and statistical analysis. Then mathematical model was statistically analyzed for fabricating desired formulation having particle size less than 200nm.

**Results:**

FT-IR, XRD reports confirmed the structural change in chitosan which lead to the formation of CsNP. For particle size, linear model was found to be best fit to explain effect of variables. Besides, high R^2^ (0.9958) defends the constancy of constructed model. Chitosan exhibited higher t-value in Pareto chart and a p-value <0.0001. Based on maximum desirability, optimization was performed and amount of variables for preparing CsNP of 180nm was predicted. The experiment was carried out with software suggested combination and particle size was found to be 176±4nm.

**Conclusion:**

Low p-value endorsed the greater dominance of chitosan on particle size. Good model adequacy and small percentage error between predicted and experimented value established the reliability of constructed model for robust preparation of CsNP.

## 1. Introduction

Cancer is the second most morbidity causing disease, and its treatment procedure is still a therapeutic and economic challenge. Even though, conventional cancer chemotherapy has improved survival rate, they also have grievous limitations [1]. For instance, chemotherapy has drug distributing nonspecifically all over human body, thus affecting both cancerous and healthy cells. This nonspecific delivery of drugs limits therapeutic dose within tumour cells while serving excess toxicity to healthy cells, tissues, and organs, consequently causing several adverse reactions including organ dysfunction, hair loss, and weakness, leading to reduced quality of life for cancer patients [2]. Tumor specificity and cutting down side effects are the significant hurdles to be crossed for successful cancer chemotherapy [3].

Methotrexate (MTX), a folic acid analogue, is a widely used chemotherapeutic agent used in the treatment of trophoblastic neoplasia, neck, and colon cancers. Dihydrofolate reductase is the principal target for MTX and inhibits intracellular folate uptake metabolism, thus intervening healthy cellular metabolism and induces apoptosis [4]. Besides that, its efficacy is often compromised by low solubility, rapid diffusion in the body, high toxicity, short half-life, and drug resistance [5]. Therefore, a need for delivery system involving MTX to enhance tumour specificity, reduce toxicity, and over drug resistance is indispensable.

Over the last decade, nanoparticles have been of much interest as they can overcome limitations of conventional chemotherapy. Nanoparticles targeting substantial tumour behaviour will provide a fruitful platform for more efficient and specific delivery of chemotherapy drugs [6]. Tumor microenvironment exhibits a unique acidic extracellular pH due to the secretion of lactic acid by anaerobic glycolytic pathway [7]. The increased CO_2_ production from the pentose pathway is another reason for acidic pH. Studies reveal acidic nature of tumour cells is very closely associated with progression of a tumour [8].

Nanoparticles can easily infiltrate through the capillary tissues, and this facilitates the efficient drug delivery to specific sites in body [9]. In particular, chitosan nanoparticles (CsNP) gained attention in the recent years due to their exceptional behavioural properties like biocompatibility, pH-responsive behaviour, and controlled drug release and can be employed as successful candidates for targeting tumour cells. Chitosan (Cs), natural polymer from crustaceans, is prepared by deacetylating chitin. Among the various methods for preparing CsNP, ionic gelation is preferred to other techniques, as it does not require extreme reaction conditions and toxic solvents [10]. Tripolyphosphate (TPP) is a nontoxic, pentavalent crosslinking agent, which in addition to chitosan solution forms rigid CsNP instantaneously through ionic interactions [11]. CsNP responds to the peculiar acidic pH conditions of tumour environment. Reports claim average pH of cancer tissue to be 6.8-7.2 and pH less than 5.5 in its cytoplasm in contrast to 7.4- 7.5 pH of healthy cells [8, 12, 13]. As a result of chitosan pH responsiveness, CsNP favours a flexible structural conformation at tumour pH and release the entrapped drug inside the cancer tissue rather than healthy cells [13, 14]. The particle size of nanocarrier plays a critical role in delivering medication to the cancer tissue. Due to Enhanced Permeation and Retention (EPR effect) observed in the perforated tumour vasculature, smaller nanocarriers of size less than 200nm can sneak past them quickly (mean epithelial pore size in tumour range from 200 to 1200nm [15, 16]. The targeted NPs also face challenges, especially lack of robust industrial preparation technique, stability, and pharmacokinetic aspects [17].

The selection and optimization of formulation and process variables are of great concern in pharmaceutical industry. Design of Experiments (DoE) emerged as a tool for better quality risk management, which commences with predefined objectives and emphasizes better understanding of product and process parameters [18]. The complete DoE process begins with deciding target product profile with superior manufacturing, efficacy, and clinical safety aspects. In fact, prior data about raw materials and experimental process is vital in designing and recognizing critical quality attributes of final product [19, 20]. Moreover, experimental procedure is designed to optimize and fabricate the final desired product. Optimization of an innovative experimental process, like those used in nanoparticles development, can lead to robust preparation technique with reproducible product with desired properties. Furthermore, factorial designs are most commonly employed method to optimize experiments and to identify which factors dominate the output and what level of these variables guide for a better and desired output [21, 22].

In this context, designing CsNP of MTX having small particle size optimized by factorial design is noteworthy. The present study aimed to investigate the significance of formulation variables in the manufacturing of MTX loaded CsNP. Furthermore, it focusses on developing a statistically reliable mathematical model for preparing CsNP of particle size less than 200nm. For this purpose, the experiment was fabricated in DoE perspective, and relation between variables and particle size was established.

## 2. Materials and Methods

### 2.1. Materials

Methotrexate was generously gifted for the study by Aeon Formulations Pvt Ltd, Puducherry. Low molecular weight (LMW) chitosan, tripolyphosphate sodium, sodium hydroxide, and glacial acetic acid were purchased from Sigma-Aldrich Chemical Co. Ltd. All other reagents used in the study were of analytical grade.

### 2.2. Experimental Design

Experimental runs were designed by Design Expert 10.0.1 [Stat Ease.Inc.] software following full factorial method. 2^3^ full factorial design was applied for examining three variables (factors) at two levels with a minimum of 8 runs. The variables screened for the study were amount of chitosan (X_1_), amount of TPP (X_2_), and amount of methotrexate (X_3_). Chitosan solutions were prepared at 2 levels of 1.0 & 2.0 mg/ml. TPP and MTX concentrations were kept at two levels as 0.8 & 1.0 mg/ml. and 30 & 60 mg, respectively. In addition to these, triplicate mid-level of variables, known as center points, were included in the study for an improved statistical significance [23]. The concentration kept for this mid-level was 1.5mg/ml, 0.9mg/ml, and 45mg for X_1_, X_2_, and X_3_, respectively. These center points will also assist in exploring the skeptical curvature effect in current design space [19]. The actual and coded experimental levels based on three-factorial two-level approach was given in Table 1, and design scheme of experiments using Design Expert software was presented in Table 2.

### 2.3. Preparation of CsNP

Instead of high molecular weight chitosan, LMW chitosan was preferred in the current study because of its relatively better solubility and colloidal stability. Molecular chitosan was dissolved in 1% w/v acetic acid solution and passed through Millipore membrane filter (pore size: 0.45*μ*m) to remove any macroscopic impurities. The pH of chitosan solution was maintained below 6.2 by adding NaOH and kept under magnetic stirrer at 800RPM. TPP and MTX were dissolved together in distilled water and maintained at pH 9.4 by adding NaOH. TPP-MTX solution (10ml) was then added to chitosan solution (25ml) in a dropwise manner to form MTX loaded CsNP and mixed at room temperature for 30 min to form stable nanocarriers. The formed CsNP were thoroughly washed with distilled water to remove the residual acetic acid. The nanocarriers were centrifuged at 10000RPM for 10 min in 3 cycles using a cooling centrifuge maintained at 4°C. The quantities of reactants were taken as prescribed by Design Expert software (Table 2). Apart from that, temperature of reaction, pH, speed of rotation, and curing time were kept constant for all experimental runs [24]. Finally, CsNP formed were freeze-dried for further characterization studies.

### 2.4. FTIR Characterization

Fourier transmission Infrared spectroscopy (FTIR) of pure methotrexate, chitosan, and prepared nanoparticle were recorded on a BRUKER spectrometer (ALPHA FT-IR). The powder was blended with anhydrous potassium bromide (KBr) in a mortar and pestle, then compressed to pellet at a pressure of 4 tons force. The IR absorbency scans were analyzed from 4000cm^−1^ to 400cm^−1^ for the change in intensity of sample peaks at a resolution of 4cm^−1^ [25].

### 2.5. X-Ray Diffraction

The powdered X-ray diffraction nature of pure chitosan and manufactured nanoparticles were recorded by X'Pert^3^ MRD (XL) operated at 30kv and 15mA with elemental copper as anode material. The specimens were analyzed at a scanning speed of 1° per min, in the range of 0°- 1000° with a sampling width of 0.010° in continuous scanning mode. The recorded graphs will aid in examining the structural changes that took place in nanoparticles with comparison to molecular chitosan [26].

### 2.6. Particle Size

Particle size measurement of prepared nanoparticle suspension was performed at room temperature using Horiba Zetasizer (SZ-100 nanoparticle). Dynamic light scattering technique was applied for particle size analysis [26].

### 2.7. Statistical Analysis

The two-level factorial design was employed to study relationship between the formulation variables (factors) and particle size (response). The following polynomial equation was used to fit the mean values of experimental data:

(1) $$\begin{array}{c} Y=\beta_{0}+\beta_{1}X_{1}+\beta_{2}X_{2}+\beta_{3}X_{3}+\varepsilon \end{array}$$

where Y represents the predicted response (particle size), *β*_0_ is intercept, (*β*_1_, *β*_2_, *β*_3_), and *ε* are main effects and model residual, respectively. X_1_, X_2_, X_3_ corresponds to independent variables. The ANOVA was performed, and P-value with 95% confidence interval was evaluated to determine the significance of each coefficient term. Similarly, lack of fit of suggested model was calculated with 95% confidence interval. To determine the fitting extent of experimental data, regression coefficient (R^2^) and adjusted R^2^ were determined. Graphs depicting predicted versus actual values and residual versus experimental runs were constructed for estimating model adequacy [19, 27, 28].

### 2.8. Optimization and Validation of Optimized Conditions

After statistical analysis, numerical optimization technique was adapted for optimizing the formulation variables for fabricating CsNP of less than 200nm in size. Based on maximum Derringer desirability function, an experimentally viable combination of variables was preferred for preparing CsNP of 180nm (less than 200nm). To investigate fidelity of optimized conditions, additional triplicate experiments were performed, and their mean was compared with predicted value. Triplicate the experiment facilitates and check the accuracy and suitability of optimized conditions in preparation of desired size range of nanoparticles by controlling the formulation parameters [29]. The optimized nanoparticle formulation was characterized for particle size analysis and SEM.

### 2.9. Scanning Electron Microscopy

The surface morphology optimized CsNPs was examined by Scanning Electron Microscopy (Quanta FEG) equipped with backscattered electron detector. The prepared nanoparticles were sputtered by Au-Pd layer over conducting aluminium stub, and high energy electron beam from a sharp high voltage tip was tunneled over specimen in the range of 15-30kV in high vacuum mode [30]. Scattered electron imaging was assessed for studying surface microstructure of nanoparticles.

## 3. Results and Discussion

### 3.1. Formation of Chitosan Nanoparticles

Chitosan is a linear crystalline polymer with intermolecular and intramolecular hydrogen bonding. In mild organic acid solutions, chitosan molecules transform into more flexible chain conformation as a result of electrostatic repulsion between the chains and due to protonation of primary amine [31]. On adding TPP, it results in spontaneous formation of nanoparticles due to formation of ionic linkage between positively charged amine groups of chitosan and negatively charged MTX and TPP ions. The particle size of formed nanocarriers is a derivative of optimum speed of rotation, pH of chitosan and drug solutions, reaction time, and curing time of experiment.

### 3.2. FTIR Characterization

**Figure 1:**
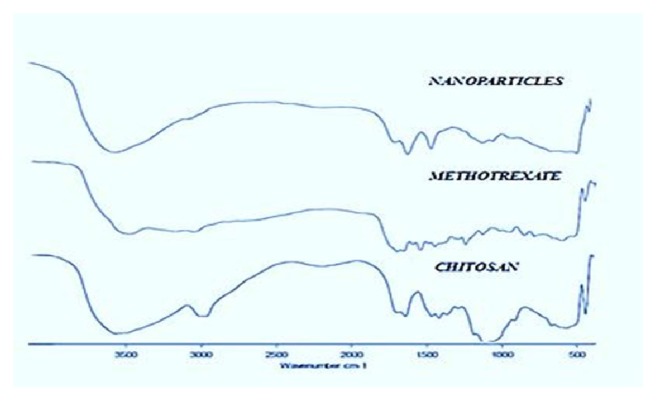
FTIR spectrum of Chitosan, MTX, and MTX loaded CsNP.

The FTIR spectra of MTX, chitosan, and chitosan nanoparticles were represented in Figure 1. Pure MTX shows characteristic transmittance vibrational peaks at 3360cm^−1^, 2954cm^−1^, 1644cm^−1^, 1603cm^−1^, 1496cm^−1^, 1404cm^−1^, and 1206cm^−1^. These bands were well demonstrated in previously reported studies and are thought to be stretches related to N-H, C-H, C=O, C-N, NH_2_ in amide and C-O group in combination with C=O moiety, respectively. In FTIR spectrum of chitosan, an intense band at 3450cm^−1^ indicates the –OH group stretching vibration.

In the CsNP, a shift in transmittance at 3450cm^−1^to 3422cm^−1^ was observed with reduced intensity, suggesting the lack of hydrogen bonding. The reduction of hydrogen bonding in the nanoparticles is due to formation of more open structure by TPP cross-linkage. Both the spectrum exhibited C-O stretching related to primary alcohol at 1084cm^−1^. The CsNP spectra represented bands at 1257cm^−1^and 1022cm^−1^ representing the presence of P=O and aliphatic P-O-C groups, clearly indicating the crosslinking with TPP. Another change in the FTIR spectrum representing nanoparticle formation is shift of bands at 1660cm^−1^ and 1595cm^−1^ to 1642cm^−1^ and 1561cm^−1^ with increased intensity, representing the change of –CONH_2_ and NH_2_ groups to protonated forms, respectively. This displacement of corresponding bands and increased intensity of NH_2_ band was also observed by other research groups in their investigation [32, 33]. This shift suggests the existence of ionic interaction between positively charged amino group of chitosan and negatively charged MTX as well as phosphate groups of TPP, thereby proclaiming nanocarrier formation.

### 3.3. XRD Characterization

**Figure 2:**
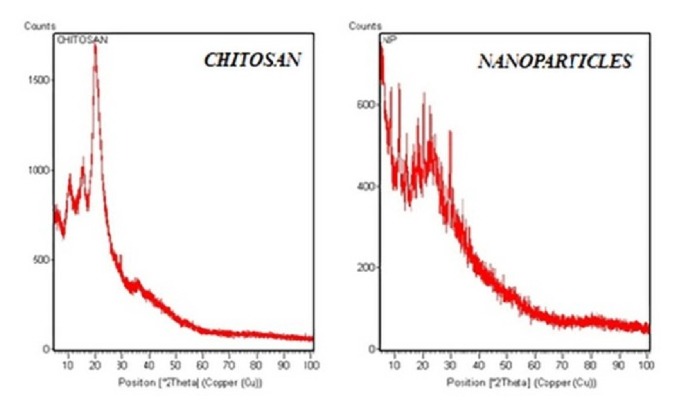
XRD graph of chitosan and MTX loaded CsNP.

XRD studies were conducted for chitosan and nanocarriers (Figure 2) at the 2*θ* range of 10^0^-30^0^; CS molecules exhibited sharp, intense peaks indicating its crystalline nature [33]. The sharp, intense peak observed at 25^0^ is due to specific hydrogen bonding of linear chitosan molecule, whereas, in XRD of nanoparticles, a wide spread of peaks with decreased intensity is noticed. The graph also depicts the lack chitosan's unique, intense hydrogen bonding peak [34, 35] and could be due to the transformation of linear crystalline molecule to a flexible chain structure by interlinked TPP molecules, thereby confirming the formation of chitosan nanoparticles.

### 3.4. Particle Size

The particle size and polydispersity index (PDI) of CsNP were analyzed by DLS technique and were depicted in Table 2. All the formulations were physically stable with a slightly yellowish appearance due to the MTX. All the formulations were moderately polydisperse in nature and in the range of 147nm to 268nm.

### 3.5. Effect of Variables

Particle size is the most important element in uptake of nanoparticles and its intracellular movement. Smaller particle size (>200nm) exhibited increased penetration in tumour tissue leading to EPR effect. Descriptive statistics of model were represented in Table 3 and results demonstrated that particle size was increased with increasing concentration of chitosan, TPP, and MTX. Calvo et al. observed some specific concentration of chitosan and TPP were highly influential over particle size [36]. This aspect was also was verified in our investigation intending to avoiding the formation of microparticles in the present experimental range. It was noticed that amount of chitosan had a huge effect on particle size of nanoparticles. This can also be witnessed in Pareto chart and also from low p-value, p <0.0001 observed in the model. To explain this effect of chitosan, it was implied that, at low concentration, intermolecular distance increases and leads to decreased cross-linking between the molecules and results in the formation of smaller nanoparticles.

The effect of MTX concentration of particle size was significant and very low p value of <0.0001 also suggests the same. When MTX concentration was increased from 30mg to 60mg, enlargement of particle size occurred which is a common observation in drug loading [37]. The effect of TPP concentration was little yet significant with a p-value of 0.003. However, the slight increase in particle size was observed as the amount of TPP was increased and could be probably due to accumulation of large TPP molecule in the nanocarrier.

### 3.6. Statistical Analysis

**Figure 3:**
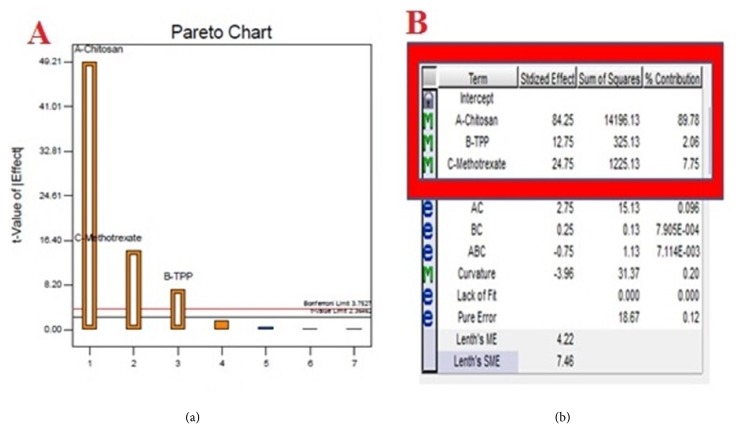
Model analysis: (a) Pareto chart and (b) percentage contribution chart.

The experimental results were analyzed by half normal plot and Pareto chart to determine the significant effects. For building the model equation, large effects were identified in half-normal plot and separated from other repeatable and small effects. The higher t-value observed in the Pareto chart endorse the selection of dominant effects altering particle size (Figure 3(a)).  Figure 3(b) depicts the individual percentage contribution of the response terms selected. The results indicated that concentration of chitosan exhibited the highest effect in altering the particle size with a percentage contribution of 89.78% as compared to other factors and was followed by the concentration of MTX and TPP with an individual contribution of 7.75% and 2.06%, respectively.

Applying multiple regression analysis, the experimental data was analyzed and fitted to various models (linear, interactive, and quadratic). The results proclaim that linear order model exhibited higher regression (R^2^), low P value, and better descriptive statistics. Hence, linear model was adopted to fit the experimental data to establish an empirical model to facilitate the interrelation between formulation variables and particle size. The final equation achieved in the coded formulation variables was given below.

(2) $$\begin{array}{c} \text{Particle size}=205.09+42.13X_{1}+6.37X_{2}+12.37X_{3} \end{array}$$

ANOVA analysis depicted that developed linear model was highly significant, as was evident from very low probability value <0.0001. The goodness of fit was checked by regression coefficient (R_2_). Here, the value of regression coefficient (R_2_ =0.9958) indicated that only 0.42% of the total variations was not explained by the adopted regression model. Besides, the difference between R_a_^2^ and R_p_^2^ was less than 0.2, which assures the reliability of model to interpolate. Furthermore, a good deal of reliability and high degree of precision of conducted experiments was indicated by low value of coefficient of variation (CV=1.50%). The adequate precision measures the signal-to-noise ratio, and a ratio greater than 4 is desirable to navigate in design space. In this case, the adequate precision was found to be 65.48, which indicates the best fitness of developed model.

**Figure 4:**
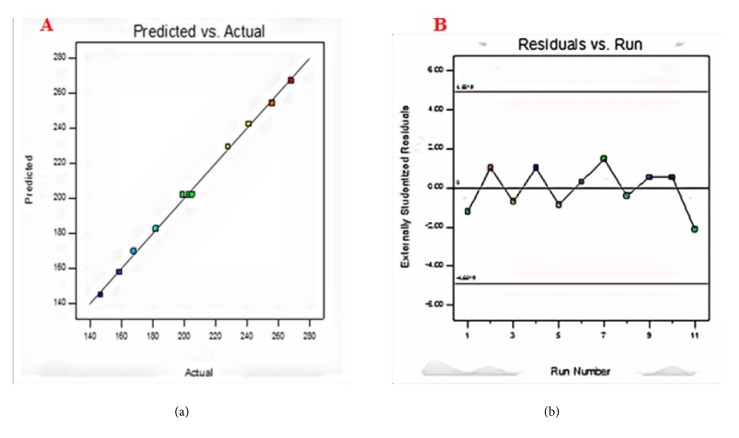
Model diagnostics: (a) predicted versus actual values; (b) residual versus run plot.

J.P.Maran et al. have reported that checking a model adequacy requires information regarding the lack of fit contained in the residuals [38]. Model diagnostic plots like predicted value versus experimental value graph helped in depicting the relationship between the experimental and predicted values and in assessing the model sufficiency. It is prerequisite to ensure if fitted linear model provides a broad approximation of the actual values and ignores small and misleading effects for optimization. In the graph drawn between the predicted versus actual values (Figure 4(a)), the data points were found to be adjacently dispersed, which indicates the minimum deviation and efficacy accord between the predicted and actual values. An internally studentized residual versus experimental runs plot was constructed to ensure the satisfactory fit of the developed model. A random trend was observed in residual vs run plot (Figure 4(b)), and all the data points fell within the range of control limits, indicating the experiments were carried out in a random manner, thereby eliminating chance of errors and ensuring adequate fit.

**Figure 5:**
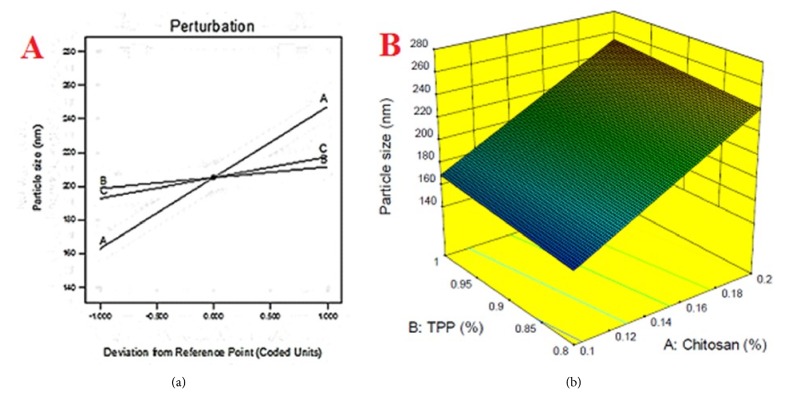
Model graphs: (a) perturbation chart; (b) 3-dimensional plot of particle size.

Intending to visualize the relationship between particle size and formulation variables, model graphs, namely, perturbation chart, contour plots, and 3D response surface, were generated, to assess the individual and interactive effects on the response. From the perturbation chart (Figure 5(a)), it was evident that the increasing the concentration of variables will increase the particle size of the formulation. The planar 3D surface diagram (Figure 5(b)) and linear curves observed that its contour region also assures the same. Moreover, the 3D plot also indicated an absence of curvature effect in the explored design space.

### 3.7. Optimization and Validation of Optimized Conditions

Regression model developed in this study was used to identify out the optimal conditions to prepare nanoparticles of 180nm. Furthermore, Derringer desirability served the purpose to decide on picking an appropriate combination of formulation variables [39]. An algebraic solution for the preparing desired nanocarriers was presented by software in the coded form and was found to be X_1_= -0.595, X_2_=- 0.005 and X_3_= 0. The corresponding experimental parameters for X_1_, X_2_, X_3_ were 1.2mg/ml, 0.899mg, and 45mg, respectively. Under these optimal conditions, the predicted particle size was 180nm. However, considering the operability of actual preparation, the optimal condition for X_2_ can be modified as 0.9 mg. To compare predicted results with experimental values, additional triplicate experiments were performed (Table 4). Under modified conditions, the particle size of experimented combination was found to be 176±4nm and well matched with predicted value 180 nm.

### 3.8. Scanning Electron Microscopy

**Figure 6:**
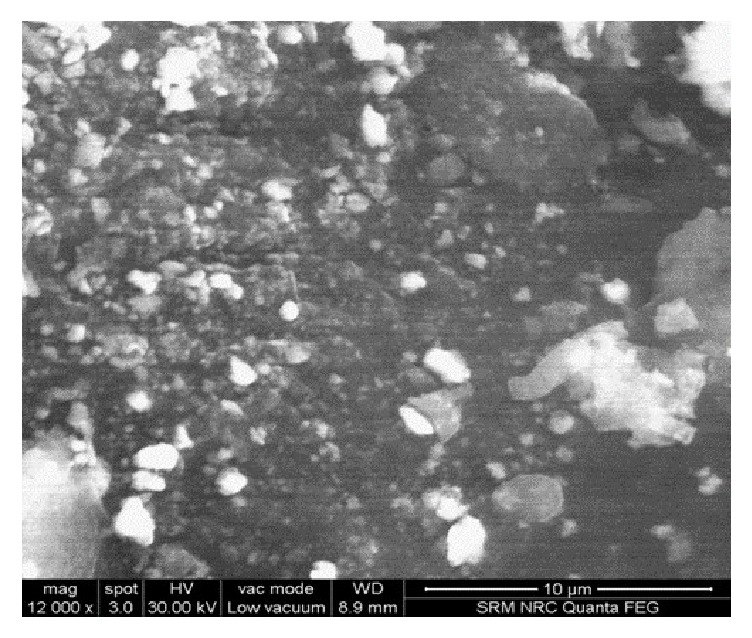
SEM micrograph of optimized MTX loaded CsNP.

Scanning Electron Microscopy, a potential surface characterizing technique, yields high resolution images of a specimen surface for a detailed topographic examination at the microscopic level. The optimized formulation was lyophilized and then examined under high voltage and the microphotograph was represented in Figure 6. To overcome limited conductivity of chitosan, low vacuum mode was adopted for the study [40]. The image depicted that particles were successfully formed with a smooth topography in ovate to spherical shapes with few nanocarrier aggregates. Aggregates were observed with shiny surfaces as well as bright edges and may be due to overlapping of particles one on the other, during either lyophilization or spreading. Bright boundaries indicate the curved nature of nanocarrier and can be related to the spherical curvature of particles. Many small-sized nanocarriers can also be observed in the field of examination with a little shadowing effect due to high voltage.

## 4. Conclusion

All the formulations were prepared randomly as per the factorial combinations by ionotropic gelation technique and evaluated for the formation of nanocarriers and particle size. The decreased intensity observed in XRD of CsNP represented the change in crystalline nature of chitosan. On the other hand, FT-IR examination revealed the interlinking of TPP and shift in hydrogen bonding of chitosan, thus conforming the formation of CsNP. The center points included in the study proved effective for analyzing particle size data, by yielding high precision value. In addition, they also authenticated the absence of curvature effect in the explored design space, certifying that linear model was best fit for the study. Furthermore, ANOVA and descriptive statistics analysis by DoE software exposed the dominance of chitosan in deciding the particle size of CsNP over other factors. However, TPP & MTX also had small, yet significant effect on nanocarrier size. Based maximum Derringer's desirability function, the desired formulation's concentration levels were originated by software, with a predicted particle size of 180nm. Experimental result, 176±4nm, was found very close to the predicted value assuring the reliability of constructed mathematical model in preparing CsNP of desired size with good reproducibility.

## Figures and Tables

**Table 1:** Coded and actual values of formulation variables.

**Factor** **(Variable)**		**Low Level**	**Mid-Level** **∗**	**High Level**
	**Coded** **Value**	**-1**	**0**	**+1**
Amount of Chitosan X_1_ (mg/ml)		1	1.5	2
Amount of TPP X_2_ (mg/ml)		0.8	0.9	1.0
Amount of MTX X_3_ (mg)		30	45	60

*∗*mid-level was followed for center point formulations.

**Table 2:** Experimental design of 2^3^ full factorial design with particle size.

**Run**	**Chitosan**	**TPP**	**MTX**	**Particle size**	**PDI**
	**(mg/ml)**	**(mg/ml)**	**(mg)**	**(nm)**	
1	1	0.8	30	147	0.465
2	1	0.8	60	168	0.682
3	1	1	30	159	0.653
4	1	1	60	182	0.504
5*∗*	1.5	0.9	45	203	0.497
6*∗*	1.5	0.9	45	205	0.468
7*∗*	1.5	0.9	45	199	0.504
8	2	0.8	30	228	0.419
9	2	0.8	60	256	0.578
10	2	1	30	241	0.423
11	2	1	60	268	0.612

*∗*= center points included in the design.

**Table 3:** Descriptive statistics of fitted linear model.

**Statistical term**	**Value**
Model p value	<0.0001
Chitosan p value	<0.0001
TPP p value	0.003
MTX p Value	<0.0001
Regression coefficient (R^2^)	0.9958
Predicted Regression coefficient (R_p_^2^)	0.9912
Adjusted Regression coefficient (R_a_^2^)	0.9940
Coefficient of variance (%CV)	1.50%
Adequate Precision	65.94

**Table 4:** Validation of optimized formulation by experimental design.

**Variable**	**Coded value**	**Actual value**
Chitosan	-0.595	1.2mg/ml
TPP	-0.005	0.9mg/ml
MTX	0	45mg

	***Predicted value***	180nm
	***Experimental value***	176±4nm

## Data Availability

The data used to support the findings of this study are available from the corresponding author upon request.
